# Association Between Antibiotic Overexposure and Adverse Outcomes in Very-Low-Birth-Weight Infants Without Culture-Proven Sepsis or Necrotizing Enterocolitis: A Multicenter Prospective Study

**DOI:** 10.1007/s12098-021-04023-w

**Published:** 2022-03-14

**Authors:** Shanshan Hou, Yonghui Yu, Yanqiu Wu, Yangyang Cao, Jinghui Zhang, Zhijie Liu, Cheng Guo, Yao Chen, Xuemei Sun, Min Li, Yanling Gao, Guoying Zhao, Shiping Niu, Zhiyuan Zhou, Yu Wang, Zhenying Yang, Lei Huang, Chengyuan Zhang, Tong Chen, Xinfeng Zhao, Xia Li, Yongfeng Zhang, Peng Zhao, Meirong Bi, Riming Zhao

**Affiliations:** 1 Department of Neonatology, Shandong Provincial Hospital, Cheeloo College of Medicine, Shandong University, Jinan, Shandong China; 2 Department of Pediatrics, Yantai Yuhuangding Hospital, Yantai, Shandong China; 3 Department of Neonatology, Shandong Provincial Hospital Affliated to Shandong First Medical University, Jinan, Shandong 250021 China; 4 Department of Pediatrics, Taian City Central Hospital, Taian, China; 5 Department of Neonatology, Qilu Hospital, Cheeloo College of Medicine, Shandong University, Jinan, China; 6 Department of Neonatology, The First Affiliated Hospital of Shandong First Medical University, Jinan, China; 7 Department of Neonatology, Affiliated Hospital of Jining Medical College, Jining, China; 8 Department of Neonatology, Shandong Provincial Hospital Affiliated to Shandong First Medical University, Jinan, China; 9 Department of Neonatology, Linyi People’s Hospital, Linyi, China; 10 Department of Neonatology, Women and Children’s Healthcare Hospital of Linyi, Linyi, China; 11 Department of Pediatrics, Dezhou People’s Hospital, Dezhou, China; 12 Department of Pediatrics, Binzhou Medical University Hospital, Binzhou, China; 13 Department of Neonatology, Zibo Maternity and Child Health Hospital, Zibo, China; 14 Department of Pediatrics, Heze Municipal Hospital, Heze, China; 15 Department of Pediatrics, Liaocheng People’s Hospital, Liaocheng, China; 16 Department of Neonatology, Taian Maternity and Child Health Care Hospital, Taian, China; 17 Department of Neonatology, Shandong Maternal and Child Health Hospital, Cheeloo College of Medicine, Shandong University, Jinan, China; 18 Department of Neonatology, W.F. Maternal and Child Health Hospital, Weifang, China; 19 Department of Neonatology, Dongying People’s Hospital, Dongying, China; 20 Department of Neonatology, Maternity and Child Health Care of Zaozhuang, Zaozhuang, China; 21 Department of Neonatology, Jinan Maternity and Child Health Care Hospital, Jinan, China; 22 Department of Pediatrics, Affiliated Hospital of Weifang Medical College, Weifang, China; 23 Department of Neonatology, Central People’s Hospital of Tengzhou, Tengzhou, China; 24 Department of Pediatrics, Jinan Central Hospital, Jinan, China; 25 Department of Pediatrics, Juxian People’s Hospital, Rizhao, China

**Keywords:** Neonate, Antibiotic use rate, Very-low-birth-weight infants, Outcome

## Abstract

**Objectives:**

To explore the associations between higher antibiotic use rates (AURs) and adverse outcomes in very-low-birth-weight (VLBW) infants without culture-proven sepsis or necrotizing enterocolitis (NEC) in a multicenter of China.

**Methods:**

A prospective cohort study was performed on VLBW infants admitted to 24 neonatal intensive care units from January 1, 2018, to December 31, 2018. AUR was calculated as calendar days of antibiotic therapy divided by total hospital days. The composite primary outcome was defined as mortality or severe morbidity, including any of the following: severe neurologic injury, bronchopulmonary dysplasia (BPD), and stage 3 or higher retinopathy of prematurity.

**Results:**

A total of 1,034 VLBW infants who received antibiotics without culture-proven sepsis or NEC were included in this study. The overall AUR of eligible VLBW infants was 55%, and the AUR of each eligible VLBW infant ranged from 3 to 100%, with a median of 56% (IQR 33%, 86%). After generalized propensity score and logistic regression analysis of 4 groups of VLBW infants with different AUR range, infants in the higher quartile AUR, (Q3, 0.57~0.86) and (Q4, 0.87~1.00), had higher odds of composite primary outcome (adjusted OR: 1.81; 95% CI: 1.23–2.67; adjusted OR 2.37; 95% CI: 1.59–3.54, respectively) and BPD (adjusted OR: 3.09; 95% CI: 1.52–6.57; adjusted OR 3.17; 95% CI: 1.56–6.57, respectively) than those in the lowest AUR (Q1).

**Conclusions:**

Antibiotic overexposure in VLBW infants without culture-proven sepsis or NEC was associated with increased risk of composite primary outcome and BPD. Rational empirical antibiotic use in VLBW infants is urgently needed in China.

## Introduction

As the world’s most populous country, China has the second largest number of premature babies in the world, with an increasing number of very-low-birth-weight (VLBW) infants in recent years [1]. Due to the high incidence and mortality of neonatal sepsis [2], clinicians are more likely to use but reluctant to halt antibiotics, resulting in widespread antibiotic overuse in VLBW infants in NICUs of China [3], which has not yet attracted sufficient attention from the government. However, increasing evidence from developed countries has suggested that unnecessary antibiotic treatment in the neonatal period may lead to colonization by multiple drug-resistant bacteria and fungemia. Prolonged antibiotic exposure in VLBW infants has been associated with an increased risk of necrotizing enterocolitis (NEC), bronchopulmonary dysplasia (BPD), retinopathy of prematurity (ROP), periventricular leukomalacia (PVL), and mortality [4]. Therefore, antibiotic overexposure may lead to unavoidable effects on VLBW infants, especially in infants without confirmed infection. However, data are scarcely reported on the association between antibiotic exposure and adverse outcomes in developing countries that have a high burden of antibiotic consumption [5]. The present study aimed to explore the current status of antibiotic use in VLBW infants among multicenters in China and to further assess the association between a higher antibiotic use rate (AUR) and short-term outcomes in VLBW infants without culture-proven sepsis or NEC to provide basic evidence for urging antimicrobial stewardship (ASP) in VLBW infants in China.

## Material and Methods

This multicenter, prospective cohort study collected data for all VLBW infants admitted to 24 participating neonatal intensive care units (NICUs) between January 1, 2018, and December 31, 2018, from the Sina-northern Neonatal Network (SNN). SNN is a regional multicenter neonatal clinical research database in China that includes preterm inpatients with birth weights (BWs) < 1500 g or gestational ages (GAs) < 32 wk. Due to more than 50% missing antibiotic data from 4 hospitals, 24 units with relatively complete data were included in this study. This study was approved by the Ethics Committee of Shandong Provincial Hospital affiliated to Shandong First Medical University and Shandong University (LCYJ: NO.2019–132). The parents of all the subjects agreed to participate in this study and signed the informed consent form, and all data were deidentified.

Infants with BW < 1500 g who were admitted to the 24 participating hospitals between January 1, 2018, and December 31, 2018, were included in the study. The exclusion criteria were as follows: infants with major congenital anomalies, infants with missing antibiotic data, infants with redirection of intensive care, infants diagnosed with culture-proven sepsis or NEC, and infants without antibiotic use.

Data on demographic information, Neonatal Acute Physiology version II (SNAP-II) scores, diagnoses, laboratory, and drug data, including drug names, classes, and dates, were collected prospectively in the database.

AUR was calculated as calendar days of antibiotic therapy divided by total hospital days [4]. The term “antibiotic” herein refers to systemic antimicrobial medications administered intravenously. The composite primary outcome was defined as mortality or severe morbidity, including any of the following: severe neurologic injury [severe intraventricular hemorrhage (IVH) (grade 3 or 4) or PVL] [6], BPD (requirement of oxygen at 36 wk postmenstrual age or at discharge) [7], and stage 3 or higher ROP [8]. The individual components of the composite primary outcome were defined as secondary outcomes.

The SNAP-II score [9] is a validated predictor of mortality in infants with a GA ≤ 32 wk that incorporates physiological derangements, mean variables of arterial pressure, body temperature, PaO2/FiO2, blood pH, occurrence of seizures, and urine output within the first 12 h of NICU admission. Small for gestational age (SGA) was defined according to the Fenton intrauterine growth curve [10]. Culture-proven sepsis was defined as a positive culture of a pathogen from blood or cerebrospinal fluid (or other sterile cavity fluids). Only clinically significant culture-proven sepsis was enrolled, which was defined as at least one positive blood or cerebrospinal culture together with clinical features consistent with systemic inflammatory response syndrome [11]. NEC was defined as a modified Bell stage ≥ IIA [12]. The NICU size was grouped based on the number of level III beds approved by the ministry and budgeted (divided by quartile, < 20, 20−29, 30−58, or > 58 level III beds).

All eligible VLBW infants were divided into 4 groups based on their AUR quartiles (Q1−Q4). Demographic data are expressed as the mean [SD] or percentages. In the univariate analysis, the Kruskal−Wallis test or chi-square test for continuous and categorical variables were used, respectively. Potential confounders and other covariates on the basis of findings in the univariate analysis were then adjusted in propensity score matching methods with multilevel treatments to control for severity of illness of patients. Balance was assessed by the generalized propensity score (GPS) [13]. Propensity scores (probability of being in groups 1, 2, 3, and 4) were then estimated, and multivariable logistic regression analyses were performed to examine the associations between outcomes and AURs, adjusting for propensity scores and NICU size, which represent the scale of the hospital. Risk was reported as odds ratios (ORs) with 95% confidence intervals (CIs). All statistical analyses were conducted using SPSS v.25.0 (SPSS Inc., Chicago, Illinois) and R software (R 4.0.3), with statistical significance evaluated using two-sided *p* values at the 5% testing level.

## Results

**Fig. 1:**
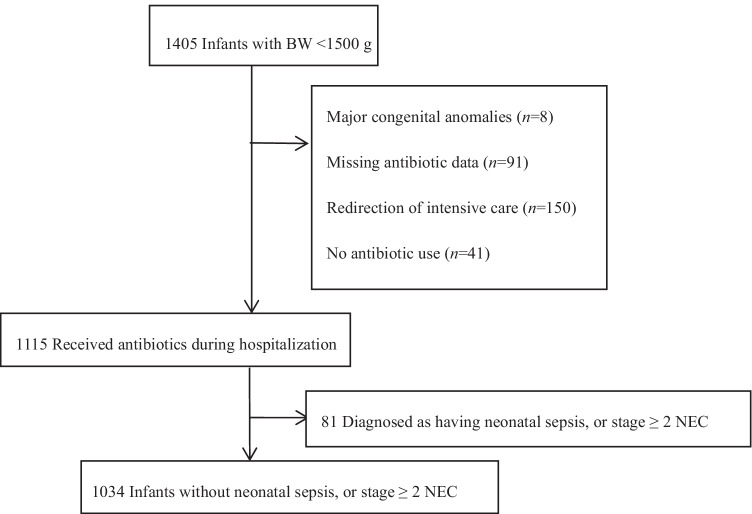
Study flow diagram. *BW* Birth weight; *NEC* Necrotizing enterocolitis

A total of 1405 VLBW infants were admitted to 24 participating NICUs in the SNN between January 1, 2018, and December 31, 2018. After excluding infants with major congenital anomalies (*n* = 8), missing antibiotic data (*n* = 91), infants with redirection of intensive care (*n* = 150), infants diagnosed with culture-proven sepsis or NEC (*n* = 81), and infants without antibiotic use (*n* = 41), the remaining 1034 VLBW infants were included in the present study (Fig. 1), none of whom started antibiotics after severe morbidity. The overall AUR of eligible VLBW infants was 55%, and the individual AUR of each VLBW infant ranged from 3 to 100%, with a median of 56% (IQR 33%, 86%).

All eligible VLBW infants were divided into 4 groups based on AUR quartiles (median 56%, IQR 33%/86%). The comparisons of infant demographic characteristics and clinical outcomes are shown in Table 1 and Table 2, respectively. Overall, infants with lower birth weight; earlier GA; lower Apgar score at 5 min; higher SNAP-II score; vaginal delivery; or the need for mechanical ventilation or vasoactive drugs were more likely to have received longer durations of antibiotics. The incidence of the composite primary outcome, BPD, and mortality were also significantly different among the 4 groups (Table 2).

**Table 1:** Demographic characteristics and outcomes of VLBW infants

	AUR quartile 1 (*n* = 254)	AUR quartile 2 (*n* = 261)	AUR quartile 3 (*n* = 268)	AUR quartile 4 (*n* = 251)	*χ*^*2*^*/Z*	*p*
AUR, range	0.03~0.33	0.34~0.56	0.57~0.86	0.87~1.00		
GA, mean (SD)	30.3 (2.0)	30.1 (2.0)	30.0 (2.2)	29.7 (2.3)	12.15	0.01
BW, mean (SD)	1230 (186)	1215 (184)	1208 (187)	1177 (230)	8.90	0.03
Male sex, *n* (%)	117/254 (46.1)	136/261 (52.1)	144/268 (53.7)	125/250 (50.0)	3.41	0.33
Cesarean delivery, * n* (%)	201/254 (79.1)	176/259 (68.0)	200/266 (75.1)	164/250 (65.6)	14.93	0.00
Multiple birth, * n* (%)	46/254 (18.1)	59/261 (22.6)	47/268 (17.5)	56/251 (22.3)	3.50	0.32
Apgar score < 7 at 5 min, *n* (%)	32/234 (13.7)	35/241 (14.5)	31/245 (15.1)	44/201 (21.9)	8.58	0.03
SGA, *n* (%)	60/254 (23.6)	55/261 (21.1)	69/268 (25.7)	53/251 (21.1)	2.25	0.52
Birth at an outside institution, *n* (%)	14/239 (5.9)	20/238 (8.4)	12/254 (4.7)	14/232 (6.0)	2.61	0.46
SNAP-II score > 20, *n* (%)	24/243 (9.8)	39/251 (15.5)	49/262 (16.0)	44/238 (18.5)	14.01	0.00
PROM ≥ 24 h, * n* (%)	42/237 (17.7)	39/222 (17.6)	39/250 (15.6)	53/221 (24.0)	5.98	0.11
Completed antenatal steroids, *n* (%)	125/223 (56.1)	108/225 (48.0)	116/232 (50.0)	108/210 (51.4)	3.15	0.37
Hypertension, *n* (%)	111/254 (43.7)	100/261 (38.4)	105/268 (39.2)	93/251 (37.1)	2.05	0.56
Mechanical ventilation, *n* (%)	99/254 (39.0)	103/261 (39.5)	113/268 (42.2)	155/251 (61.8)	32.36	0.00
Vasoactive drugs, * n* (%)	7/254 (2.8)	19/261 (7.3)	26/268 (9.7)	28/251 (11.2)	16.27	0.00

The following information was missing: sex, 1 infant; birth at an outside institution, 11 infants; cesarean delivery, 5 infants; Apgar score < 7 at 5 min, 113 infants; PROM ≥ 24 h, 104 infants; completed antenatal steroids, 144 infants; SNAP-II score > 20, 40 infants

*BW* Birth weight (g); *GA* Gestational age (wk); *PROM* Premature rupture of membrane; *SGA* Small for gestational age

**Table 2:** Clinical outcomes of VLBW infants

	AUR quartile 1 (*n* = 254)	AUR quartile 2 (*n* = 261)	AUR quartile 3 (*n* = 268)	AUR quartile 4 (*n* = 251)	*χ*^*2*^	*p*
BPD, *n* (%)	13/254 (5.1)	34/261 (13.0)	41/268 (15.3)	32/251 (12.7)	14.80	0.00
Severe neurologic injury, *n* (%)	69/254 (27.3)	72/261 (27.6)	94/268 (35.1)	67/251 (26.7)	6.07	0.11
ROP (≥ stage 3), * n* (%)	4/254 (1.6)	6/261 (2.3)	6/268 (2.2)	5/251 (2.0)	0.42	0.94
Mortality, *n* (%)	1/254 (0.4)	5/261 (1.9)	4/268 (1.5)	71/251 (28.3)	196.62	0.00
Composite primary outcome	76/254 (30.3)	101/261 (38.6)	120/268 (44.8)	138/251 (55.0)	34.54	0.00

The composite primary outcome was mortality or major complications, including severe neurologic injury, bronchopulmonary dysplasia, and stage 3 or higher retinopathy of prematurity

*BPD* Bronchopulmonary dysplasia; *ROP* Retinopathy of prematurity

**Fig. 2:**
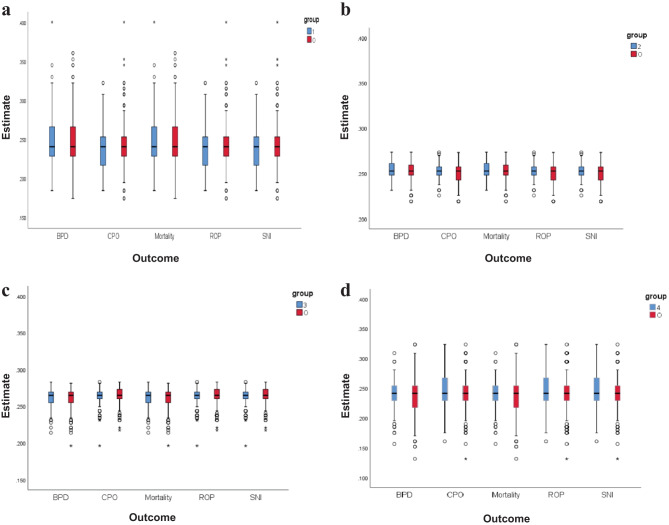
**a –d** Normalized difference for GPS for each group. Rectangles indicate the interquartile range and median. Lines above or below the box extend farther by 1.5 times the interquartile range. Dots indicate extreme outliers. *BPD* Bronchopulmonary dysplasia; *CPO* Composite primary outcome; *O* the other groups; *ROP* Retinopathy of prematurity; *SNI* Severe neurologic injury

GA, sex, Apgar score at 5 min, SNAP-II score, cesarean delivery, mechanical ventilation, and vasoactive drugs were adjusted for propensity score matching methods with multilevel treatments. For each group, the normalized difference for GPS were calculated for that group and plotted a box-whisker plot of GPS for infants in each group and a box-whisker plot of GPS for the remaining infants in the same figure (Fig. 2 a−d). Group 1 (Fig. 2 a), group 2 (Fig. 2 b), group 3 (Fig. 2 c), and group 4 (Fig. 2 d) represented AUR quartile 1, AUR quartile 2, AUR quartile 3 and AUR quartile 4, respectively, while group O represented the rest of the infants. The horizontal axis represents the outcome, and the vertical axis represents the estimated GPS. As Fig. 2 a−d shows, the median GPS of the two groups was very close for each outcome in each figure. The interquartile range of the two groups for BPD and mortality was also very close in Fig. 2 a−d, while the 75% quantile or 25% quantile of the two groups were very close for the composite primary outcome, ROP and severe neurologic injury in Fig. 2 a−d. After adjustment for propensity score and NICU size by multivariable logistic regression, infants in the highest quartile AUR (Q4) had higher odds of composite primary outcome, BPD, and mortality than those in the lowest AUR (Q1). The higher quartile AUR (Q3) also had higher odds of composite primary outcome and BPD than those in the lowest AUR (Q1) (Table 3).

**Table 3:** Analyses of antibiotic exposure and outcomes by generalized propensity score methods and multivariable logistic regression

OR (95% CI)				
	AUR quartile 4 (Q4)	AUR quartile 3 (Q3)	AUR quartile 2 (Q2)	AUR quartile 1 (Q1)
Composite primary outcome	2.37 (1.59, 3.54)	1.81 (1.23, 2.67)	1.38 (0.93, 2.06)	1
Severe neurologic injury	0.85 (0.55, 1.31)	1.36 (0.90, 2.03)	0.89 (0.58, 1.36)	1
ROP (≥ stage 3)	1.31 (0.34, 5.04)	1.43 (0.39, 5.21)	0.92 (0.22, 3.82)	1
BPD	3.17 (1.56, 6.57)	3.09 (1.52, 6.57)	2.92 (1.42, 6.04)	1
Mortality	63.13 (8.54, 466.93)	5.07 (0.58, 44.12)	5.11 (0.59, 44.37)	1

The composite primary outcome was mortality or major complications, including severe neurologic injury, BPD, and stage 3 or higher ROP

*BPD* Bronchopulmonary dysplasia; *ROP* Retinopathy of prematurity

## Discussion

In this multicenter prospective cohort study of China, it was found that the overall AUR of VLBW infants who received antibiotics without culture-proven sepsis or NEC was 55%, and the median AUR of each VLBW infant was 56% (IQR 33%/86%), which were both much higher than those in developed countries [4, 14]. More importantly, the higher AUR was significantly associated with an increased risk of composite primary outcome and BPD in VLBW infants without confirmed infection.

Over half of the VLBW infants in the present study were treated with antibiotics for more than half of the patient's length of stay (AUR > 56%). However, the median AUR of VLBW infants was only 17% in a multicenter study of the Canadian Newborn Network (CNN) [4]. Data in the present study were significantly higher than that of CNN but similar to the multicenter report in Hunan Province in China [3], reflecting widespread antibiotic overuse in VLBW infants in China. Inappropriate antibiotic use with initiation and continuation was more common in Chinese hospitals than in developed countries in a previous study [15]. The latest guidance from the American Academy of Pediatrics for preterm infants [16] suggested that physicians need to consider the risk/benefit balance of empirical antibiotic therapy for infants at low risk for early-onset sepsis (EOS). Empirical antibiotic therapy for infants with a high risk for EOS should be discontinued 36 to 48 h after culture unless there is clear evidence of infection. Given the large population of patients, limited newborn ward capacity and lack of full-time specialized staff [17], accompanied by a high incidence of multidrug-resistant bacteria in China [18], it may not be feasible/safe to withhold antibiotics if the sickness scores and antenatal management strategies are completely different from those in the Western world. Therefore, clinicians in China are prone to empirically use antibiotics after birth, even in VLBW infants at low risk for EOS. In addition, due to the lack of consciousness of antibiotic timing when ruling out sepsis, the duration of antibiotic courses was significantly longer than those reported in developed countries [15]. In 2011, the China Ministry of Health (MOH) began implementing ASPs in hospitals for proper antibiotic use [19], but there were no systematic improvement strategies for neonatal ASPs. Common effective ASP interventions, such as sepsis risk calculators and automatic stop systems for 48-h empiric antibiotics [20], are still rare in China. Given the increase in medical disputes in recent years, clinicians may be inclined to the overuse of antibiotics to reduce the possibility of a lawsuit due to malpractice (self-protective treatment) [21]. All of the above factors may have contributed to antibiotic overexposure in the authors' multicenter study.

In the analysis of the associations between different AURs and adverse outcomes in VLBW infants, severe neurologic injury, BPD, and ROP were included in the primary outcome since their chronological sequence usually occurs after the median age of infection in preterm neonates. The authors found increased adjusted ORs of the composite primary outcome and BPD in VLBW infants in the higher AUR quartile and significantly increased ORs of mortality in VLBW infants in the highest AUR quartile compared to the lowest quartile. The findings of the present study were similar to those reported in the literature. Cantey et al. [22] reported that each additional day of antibiotic therapy was associated with an increased risk and severity of BPD. A CNN study [4] also showed that mortality, BPD, and ROP were associated with the duration of exposure to antibiotics. At the individual level, antibiotic use, both perinatally and postnatally has been linked to disruptions in the microbiome [23, 24]. Microbiota in the gut contribute to maturation of the intestinal immune system in early life, the establishment of an efficient barrier to luminal antigens and bacteria, and the regulation of proinflammatory and anti-inflammatory immune responses [25, 26]. In a piglet model, treatment with antibiotics reduced the diversity of gut microbiota and reduced the expression of a large number of immune-related genes [27]. Greenwood’s survey [28] showed a higher percentage of enterobacter colonization and lower bacterial diversity in preterm infants who received 5–7 d of empirical antibiotics than in those not exposed or exposed to shorter courses. Because of the association of the pathogeneses of PVL, ROP, and BPD with systemic inflammation, the disruption of gut microbiota due to antibiotic overuse may lead to an increasing incidence of these diseases and mortality. Furthermore, antibiotic exposure during early life can also lead to long-term adverse outcomes, such as asthma, inflammatory bowel disease, and childhood obesity [6]. However, the authors do not have medium- or long-term health outcome data of infants with varying antibiotic exposures.

As far as the authors' know, this study is the first multicenter, observational cohort study with a large sample to investigate the real-life situation of antibiotic overexposure and its adverse outcomes in VLBW infants in China. Antibiotic overuse has increasingly become a serious problem worldwide, with potential for the emergence of multidrug-resistant bacteria and even ‘superbugs’. However, this study presents only an overview of antibiotic use in VLBW infants and does not further represent data about the reasons for antibiotic overuse. The authors are planning to develop multicenter ASP and lead a deep survey about reasons for inappropriate antibiotic use and differences in antibiotic use strategies among NICUs. Another limitation of this study is confounding by indication as an observational study, which could lead to a false association between adverse outcomes and treatment. In this study, GPS was used to address the limitations of confounding bias of multilevel AUR. The idea of GPS for multilevel treatments is relatively new, but it is more advantageous than simple multivariable logistic regression due to the weak nonconfoundedness assumption that assignment to a certain treatment level is independent of the potential outcome given the GPS [29]. In addition, due to the lack of current standardized definitions of pneumonia in preterm infants and the lack of a uniform consensus definition for culture-negative sepsis, the true burdens of these infections could not be identified and evaluated. Furthermore, there was a risk for observer effects in the prospective study, as the clinicians in the authors' centers were aware of the study. However, the present study was not an intervention study, and there was no significant change in the frequency of antibiotic use between the prospective period and before, suggesting that any observer effect was minimal. Finally, the types of antibiotics or their causative effects on neonatal complications were not studied. The authors are planning to further study the adverse effects of narrow-spectrum and broad-spectrum antibiotics on neonatal outcomes.

## Conclusions

Antibiotic overexposure in VLBW infants without culture-proven sepsis or NEC was common and was associated with an increased risk of composite primary outcomes and BPD, which is a serious health issue in China. More efforts should be focused on the development and implementation of antimicrobial stewardship programs that can help reduce the burden of antibiotic use in countries where bacterial resistance is rising, such as China.
